# No apparent association between lecture attendance or accessing lecture recordings and academic outcomes in a medical laboratory science course

**DOI:** 10.1186/s12909-020-02066-9

**Published:** 2020-06-30

**Authors:** Sheila Anne Doggrell

**Affiliations:** Faculty of Health, Queensland University of Technology, Brisbane, QLD 4002 Australia

**Keywords:** Academic outcomes, Lecture attendance, Lecture recordings, Lecture slides, Medical laboratory science students, Survey

## Abstract

**Background:**

The effect of availability of lecture recordings on academic outcomes is not clear and it is not known whether these recordings change the association between lecture attendance and academic outcomes. Few surveys of lecture attendance or lecture recordings use by students are linked to academic outcomes. The aims were (i) to determine any association between lecture attendance and academic outcomes for students who had access to lecture recordings, (ii) to determine any association between accessing lecture recordings and academic outcomes and (iii) to use a survey to determine why students attend lectures and/or access lecture recordings in a course in medical laboratory science.

**Methods:**

Consenting students signed in when attending lectures and/or completed an online survey. Pearson’s correlation coefficients were calculated to determine whether there was an association between attending lectures or accessing lecture recordings and academic outcomes.

**Results:**

Consent rates were high for both the sign-in (90%) and survey (64%). The main findings were that in 2017 and 2018: (i) the average lecture attendance was 39 and 27%, respectively, (ii) there was no association between lecture attendance and academic outcomes, (iii) there was no association between accessing lecture recordings and academic outcomes. Survey respondents were almost equally divided between those attending lectures weekly, sometimes or not. Reasons for attending lectures included greater perceived learning and interaction with staff and other students, while reasons for not attending related to inconvenience or other commitments. Lecture recordings were accessed to clarify, revise or catch up on content, or as an alternative to attending lectures. One-third of students provided additional feedback on accessing lecture recordings, and the most common themes were ‘flexibility’ and ‘useful’. Lecture slides (PowerPoints), independently of lecture recordings, were used extensively by the students.

**Conclusions:**

From this study, it does not seem that either lecture attendance or accessing lecture recordings are major determinants of academic outcomes for most students. As students vary in their lecture attendance and use of online resources including lecture recordings and lecture slides, academic staff should continue to provide a range of resources for students.

## Background

Previous academic performance and study skills were the first identified predictors of academic performance at university [1]. Subsequently, other factors have been identified as predictors including psychological predictors (e.g. commitment and satisfaction with university), cognitive appraisal, and demographics (e.g. employment responsibilities, student workload [1]). Attending lectures is also considered by many teachers to be a predictor of performance. This was tested in a meta-analysis of 68 studies of US college students in 2010, which showed that students who attended lectures more frequently obtained better grades [2]. The meta-analysis also showed that attendance was a stronger predictor of performance than other known predictors including college entry scores, study habits and study skills [2].

In the 2010 meta-analysis, the positive relationship between lecture attendance and academic performance was also observed for the subgroup of 11 studies of students studying science [2]. The individual studies for students of the biological sciences included in the meta-analysis and subsequent studies of these students have mostly shown a positive association between lecture attendance and academic outcomes [3–21], but some have not [22, 23].

As no information on the availability of lecture recordings to students is presented in the meta-analysis of Credé et al., 2010 [2], it is not clear whether this availability changes any association between lecture attendance and academic performance. As most (40) of the 68 studies combined in the meta-analysis were before or in 2000, they probably do not relate to the current teaching/lecture recording environment, where lecture recordings are available to students. The availability of lecture recordings probably reduces lecture attendance [24, 25].

In the biological sciences, studies of any association between lecture recordings usage and academic outcomes have yielded diverse results. For nursing students studying anatomy, physiology and/or microbiology, lecture recording usage was associated with higher [26] or lower course [27] grades. Other studies have shown no effect of lecture recording usage on grades/attainment (BSc students [25], medical science students [28, 29], pharmacy students [30]). For medical students, there was no association in seven courses [31] and a negative association in two courses [31, 32], and for dental students there was no association in six courses and a negative association in one course [23].

Surveys of biological science students, linked to academic performance, about their lecture attendance indicate that the major reasons given for non-attendance at lectures were lectures being too early in the day, followed by a large gap between classes, too few timetabled hours, lack of sleep, and the poor quality of lecturing [33] or preparing for another examination, followed by lack of interest, lecturer’s teaching style, and availability of lecture material [34]. One survey of students studying pharmacology, linked to academic outcomes, reported that the main reason for accessing lecture recordings was for revision, followed by clarification, having missed the lecture, and unsuitable timetabling of lectures [10].

These studies have not considered whether the availability of lecture recordings altered the relationship between lecture attendance and academic outcomes. It is also not known whether students who have low lecture attendance, but access lecture recordings, have improved performance, compared to those who do not attend or access recordings. The present study addressed these issues with participants who were third year undergraduate medical laboratory science students in a diagnostic endocrinology course. These students are being trained to work as medical laboratory scientists in pathology laboratories interpreting laboratory tests on medical specimens to assist with the diagnosis of medical conditions. To the author’s knowledge, there have been no studies of lecture attendance or lecture recordings usage and associations with academic outcomes for these students.

The first aim of this study was to determine any association between lecture attendance and academic outcomes for students who had access to lecture recordings. This was to test the hypothesis that the positive relationship between lecture attendance and academic outcomes is weak in the presence of lecture recordings. The second aim was to determine any association between accessing lecture recordings and academic outcomes to test the hypothesis that there is a positive association between accessing lecture recordings and academic outcomes. A third aim was to explore through an online survey why students attend lectures and how they use lecture recordings and other resources.

## Methods

The Bachelor of Medical Laboratory Science degree at Queensland University of Technology (QUT) considers specialised concepts in biochemistry, haematology, immunology, microbiology, pathology, transfusion and transplantation science, quantitative medical science, quality assurance systems, and health informatics. The Diagnostic Endocrinology course is a specialised biochemistry course taught as part of this degree. It is a third-year level, 12 credit point course (96 credits/year is full-time study), which was delivered similarly in 2017 and 2018. There is no recommended textbook, but students are provided with a list of reference texts. The course had 2 h of lecturing/week over 13 weeks, which were made available via Blackboard as recordings (Echo 360; voice and lecture slides). The course was supported by a weekly one-hour tutorial, and 2 h of laboratories/week for weeks 1–10, followed by seminars/student presentations for weeks 11–13. The marks were 40% for the examination, which is a mixture of multiple-choice questions and short answer questions, restricted to lecture content, and 60% for ongoing assessment. The ongoing assessment was 40% for laboratories and 20% for a team oral presentation. The laboratory marks were 30% for weekly reports and quizzes and 10% for a practical examination. The laboratories were related to the lecture content. The oral presentation was an in-depth study of a topic, discussed briefly in the lectures, and had an individual component.

The author was not involved in any aspect of the running or teaching of the Diagnostic Endocrinology course. As it seemed likely to the author that the number of students who would undertake the survey associated with this research would be lower than the numbers who would consent to sign the attendance register, consent and ethic approval was sought separately for these. This was to maximize participation and minimise selection bias in the part of the research involving signing the attendance register. Thus, two ethical approvals for this research were obtained from the Human Research Ethics Committee at Queensland University of Technology; Ethics Approval Number 1700000690, which was for the “sign-in”, and 1700000873 which was for the “survey” component of the study. During the laboratories in week 1 and 11, written consent was sought from the students by the author to undertake the “sign-in” and “survey” components, respectively.

### Sign-in component

The “sign-in” component was predominantly to determine whether attendance or non-attendance at lectures affected academic outcomes. Thus, the students were asked to consent to sign an attendance register at each lecture, and for permission to link this data to their academic outcomes. A list of students who had consented to participate was prepared, and then circulated at the lectures from week 2 onwards, to allow attending students to sign. From the list, the percentage of attending students/week was determined and averaged, and for each consenting student, the number of lectures attended was collated.

Grades for participating students were collated and averaged. Like most Australian universities, passing grades at QUT are 4 (overall mark, 50–64%), 5 (65–74%), 6 (75–84%) and 7 (≥ 85%). Academic outcomes measured were the overall mark, marks for the examination, and for the ongoing assessment. These marks for the individual components were totalled and the total expressed as a percentage and then the percentages were averaged. Regression line analysis of marks vs lecture attendance was undertaken, and Pearson’s correlation coefficients were calculated to determine whether there was an association. In addition, the marks for those participants attending no vs some lectures, < 33% vs ≥ 33% attendance, < 50% vs ≥ 50% attendance, < 70% vs ≥ 70% attendance, were compared by Student’s unpaired t-test.

### Survey

The survey explored factors affecting students’ lecture attendance and lecture recording usage, and determined any association between self-reported lecture attendance, lecture recording usage and academic outcomes. Consenting students were asked and reminded to complete the online survey (Supplementary online information 1), available via a link in the course Blackboard page. Extra questions were added to the survey in 2018 (Supplementary online information 2). The survey was available after consent was sought in week 11 until the day prior to the final examination.

The survey asked the students to provide their student numbers (IDs) to allow correlation between self-reported lecture attendance or accessing lecture recordings and marks. Overall marks were compared between the sign-in and survey components of the study.

There were a series of questions in the survey relating to lecture attendance, lecture recordings, resources and lecture slide use, and students were asked to tick all that apply. Not all students who undertook the survey answered all the questions. For each option, the number and percentage of students giving that answer is presented. The final question on the survey was “Please include any additional comments or feedback you have on the use of lecture recordings as a leaning tool”, and the responses were evaluated using thematic analysis [35].

Pearson’s correlation coefficients were calculated between a points measure of student engagement derived from the survey (online supplementary information 1), for self-reported lecture attendance, lecture recording usage and/or lecture slide usage, and outcomes (overall mark, examination and ongoing assessment): 0 for no attendance/access lecture recordings, 1 for sometimes and 2 for weekly or most weeks. For lecture slide use outside of lecture recording access, there were 5 options and 0 points given for the no use option, and 0.5 points for each option with a positive response (online supplementary information 2). Engagement was a combination of points for lecture attendance, accessing lecture recordings and lecture slide use.

### Statistics

Percentage attendance of students in weeks 2–5 and weeks 6–13 were compared by Student’s paired t-test. Mean values for percentage attendance and academic outcomes (overall mark, examination, ongoing assessment) ± standard deviation (SD) were determined. Academic outcomes between attendance groups were compared by Student’s unpaired t-test and *P* values of less than 0.05 considered significantly different. Association between lecture attendance, lecture recording use, lecture slide use, combinations of these, and academic outcomes were analysed by Pearson’s correlation coefficient (r) and significance (*P* value) were determined using Microsoft Excel.

## Results

The research was undertaken in semester 2 of 2017 and 2018. In 2017, 48 students were enrolled at the start of semester, and in 2018, 69 students.

### “Sign-in”

#### Lecture attendance and its relationship to academic outcomes

In 2017 and 2018, 41 (85%) and 64 (93%) students, respectively, consented to undertake the attendance sign-in at lectures. None of the consenting students withdrew during the semester, but there were four failures of five or less percentage points (one in 2017, three in 2018).

#### Lecture attendance

Percentage attendance was not measured in week 1, as this was prior to the consent collecting process. In week 8, there was no face-to-face lecture, and the final week was a revision session in lieu of a lecture. In 2017, week 3 was a public holiday and in week 6 attendance data were inadvertently not collected.

**Fig. 1:**
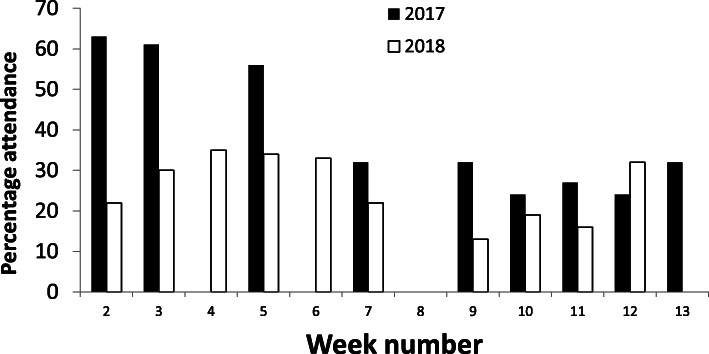
Percentage of students attending the lectures plotted against the week number in semester

The average percentage lectures attendance/student was 39% ± 34 and 27% ± 31 in 2017 and 2018, respectively. Attendance was higher at the start of semester than later (Fig. 1): 2017, weeks 2–5, 61% ± 42, weeks 6–13, 29% ± 34, *P* < 0.05; 2018, 31% ± 36 vs 24% ± 31, *P* = 0.22.

#### Lecture attendance and academic outcomes

**Fig. 2:**
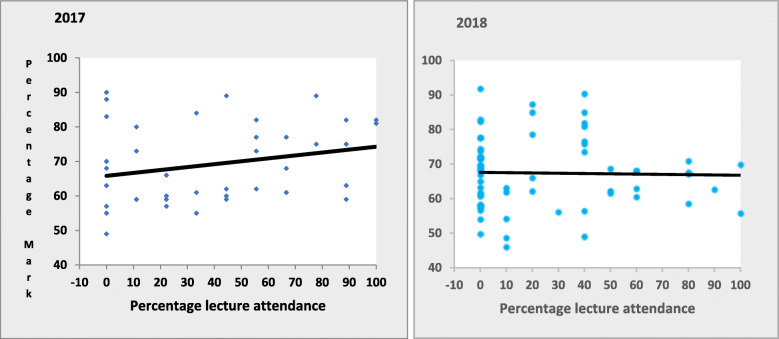
Regression line analysis of the association between overall course mark and percentage of lectures attended in 2017 (Left) and 2018 (Right)

Regression line analysis of overall mark versus lecture attendance revealed no outliers and no significant association in both 2017 and 2018 (Fig. 2). Similarly, there was no significant association between lecture attendance and the examination or ongoing assessment in both 2017 and 2018 (Table 1). In a secondary analysis, divisions of students into those who attended no vs at least one lectures, < 33% vs ≥ 33% attendance, < 50% vs ≥ 50% attendance, < 70% vs ≥ 70% attendance, did not show any association between lecture attendance and academic outcomes (Supplementary Table 1).

**Table 1:** Regression line analysis of academic outcomes vs lecture attendance from sign-in

	2017 (*n* = 41)		2018 (*n* = 64)	
Academic outcome	Pearson’s correlation r	***P*** value	Pearson’s correlation r	***P*** value
Overall mark	0.247	0.119	−0.024	0.849
Examination lecture content	0.280	0.076	0.052	0.685
Ongoing assessment (total)	0.186	0.242	−0.185	0.143
(i) Laboratories	0.238	0.133	−0.194	0.125
(ii) Oral presentation	0.048	0.762	−0.016	0.902

### Survey

In 2017 and 2018, 32 and 43 students, responded to the survey (67 and 62% of enrolled students, respectively.

#### Overall marks of students who participated in the survey vs sign-in

There was no difference between the overall marks for the students who participated in the survey versus those who undertook the attendance sign-in study. The overall marks for the students who responded to the survey were 69% ± 11 (32) and 68% ± 11 (43) which were not significantly different from the values for the sign-in component, 69% ± 11 (41) and 67% ± 10 (64), in 2017 and 2018 respectively.

#### Survey responses from students who attended lectures regarding reasons for attendance and accessing lecture recordings

In 2017, 12 students from the survey reported attending lectures in most weeks (38%), 10 sometimes (34%) and 10 not at all (31%). In 2018, similar percentages reported attending most weeks (16 students, 37%), sometimes (12 students, 28%) and not at all (15 students, 35%). The 22 (2017) and 28 (2018) students who attended most weeks or sometimes were asked additional questions about why they attended lectures and why they accessed lecture recordings as well as attending lectures with multiple possible answers (tick all that apply). As results were similar in 2017 and 2018, responses from the 2 years were combined (Table 2).

**Table 2:** Survey responses from students who attended lectures weekly or sometimes

**Why did you choose to attend lectures?**	*n* = 50, rp = 50
*I think I learn more by attending*	40 (80%)
*It allows for interaction with course staff and/or students*	35 (70%)
*I am concerned that recordings may not be complete or the technology for recording may fail*	30 (60%)
*To catch up with my friends*	16 (32%)
*It is good to be seen to be attending*	14 (28%)
*I like to see the lecturer’s gestures and expressions*	14 (28%)
*I think my results will be better if I attend*	13 (26%)
**Why did you access the recordings as well as attending the lectures?**	*n* = 43, rp = 43
*Clarify difficult concepts*	37 (86%)
*Revise lecture concepts for assessment purposes*	35 (81%)
*Catch up on lectures I missed*	34 (79%)
*Reinforce and revise concepts on a regular basis*	22 (51%)
*I find it hard to concentrate in the lecture theatre*	13 (30%)

*n* is the number of students in this group, rp is the number of responders in the group

The students who attended lectures most weeks or sometimes were asked if they also accessed the lecture recordings, and most did (Table 3). The students who reported attending lectures were asked if they found it useful to hear and see the lecture content again after attending the scheduled lectures, and most considered it to be very useful (64%) quite useful (36%) and not useful (0%) for study and increasing understanding of concepts.

**Table 3:** Use of lecture recordings by students who attended or did not attend lectures

Students who attended lectures	Most weeks or sometimes	Not at all
**How often did you access lecture recordings?**	*n* = 50	*n* = 25
Most weeks	20 (40%)	8 (32%)
Sometimes	23 (46%)	16 (64%)
Not at all	7 (14%)	1 (4%)

Sometimes includes “*Sometimes*”, “*Sometimes – I listened to recordings from Blackboard*”, “*Sometimes I downloaded recordings from Blackboard to my personal device*”, “*Yes – every few weeks*”, “*Yes at the end of semester before the final exam*”

Answers are number of students (percentage)

#### Survey responses from students who did not attend any lectures regarding reasons for non-attendance and accessing lecture recordings

Survey responses from students who did not attend any lectures were similar in 2017 and 2018, so are combined in Table 4. Most of the students who did not attend lectures accessed lecture recordings (Table 4). The most common reason given by the students (77%) who did not attend the lectures was because it was too early (8 am). Students also did not attend lectures due to the inconvenience of having too far to travel, and not having enough other timetabled classes or because of other commitments (Table 4). The students who did not attend lectures, were also asked why they accessed lecture recordings instead of attending lectures, and the reasons given were similar to those for not attending lectures (Table 4).

**Table 4:** Survey responses from students who did not attend any lectures

**If you did not attend lectures, why not?**	*n* = 25, rp = 22
*I don’t like the lecture time – it was too early*	17 (77%)
*Too far to travel for lecture*	11 (50%)
*When assessment tasks were due, they took preference over lecture attendance*	11 (50%)
*I had too few timetabled classes that day and didn’t want to come in for just those*	9 (41%)
*Other personal commitments made it difficult to get to lectures*	8 (36%)
*Work commitments made it difficult to get to lectures*	8 (36%)
*I don’t consider the lecturer adds to the material given on the PowerPoints*	5 (23%)
*I don’t like the lecture theatre environment*	3 (14%)
*Prefer online learning environment*	1 (5%)
**If you did not attend lectures, why did you access the recordings instead of attending lectures**	*n* = 25, rp = 25
*I didn’t like the lecture time – it was too early*	22 (88%)
*When assessment tasks were due, they took preference over lecture attendance*	14 (56%)
*I had too few timetables classes that day and didn’t want to come in for just those*	12 (48%)
*Too far to travel for lectures*	12 (48%)
*Work commitments made it difficult to get to lectures*	9 (36%)
*Other personal commitments made it difficult to get to lectures*	8 (32%)
*I prefer the flexibility of online recordings*	6 (24%)
*I don’t like the lecture theatre environment*	4 (16%)
*I don’t consider the lecturer adds to the material given on the PowerPoints*	4 (16%)
*I chose to rely on cramming the lecture material at the end of semester using the lecture recordings*	2 (8%)
*Prefer online learning environments*	1 (4%)

*n* is the number of students in this group, rp is the number of responders in the group

Answers are number of responding students (group percentage)

#### Use of other resources

In the survey, the students were asked which resources they used for assessment purposes, other than attending lectures and accessing lecture recordings, and 45 students answered this question. The most common resource used was *Teaching resources on Blackboard* e.g. *PowerPoint slides* (93%), followed by *Practical information and reports* (69%), *Reference books* (33%) and *Other students’ notes* (13%).

Extra questions were added to the survey in 2018 to ascertain whether the students were using the lecture slides separately from lecture recordings. The first question was “*Other than as part of lecture recordings, do you use the hard copies of the provided PowerPoint slides or PowerPoint slides online”* and most students responded that they did (34 out of the 43 respondents; 79%). Most of students who used lecture slides separately from lecture recordings did so to study prior to assessment or examinations (88%, Table 5). However, this use was not associated with better academic outcomes as the overall mark was 67% ± 12 (30) for the students who used the lecture slides outside of lecture recordings prior to assessment or examination, compared to 69% ± 11 (13) for the students who did not use the lecture slides in this way. Some of the students used the lecture slides instead of attending lectures (42%) or to study instead of listening to lecture recordings (27%, Table 5). Indeed, there was overlap between these categories with 24% of students using lecture slides instead of attending lectures or listening to lecture recordings.

**Table 5:** Use of lecture slides (PowerPoints) outside of lecture recordings

	2018
*To study prior to assessment or examination*	30 (88%)
*To study during lectures*	24 (71%)
*To study instead of attending lectures*	14 (42%)
*To study instead of listening to lecture recordings*	9 (27%)
*To study prior to lectures*	8 (24%)

Answers are number (percentage) of *n* = 34 students indicating how they used the lecture slides separately from lecture recordings

#### From the survey, lecture attendance or accessing lecture recordings or slides use or the combination (engagement) and academic outcomes

As described in the methods, 0 points were allocated for ‘no’ attendance or recording access response, 1 for ‘sometimes’ and 2 for ‘most weeks’ or ‘weekly’. In 2017 and 2018, there was no significant association between the points for lecture attendance or accessing lecture recordings or the combination of lecture attendance and accessing lecture recordings and the academic outcome of overall mark, examination or ongoing assessment (Supplementary Table 2). In addition, in 2018, there was no significant association between lecture slide use or the combination of lecture attendance, accessing lecture recordings and lecture slide use (engagement) and overall mark, examination or ongoing assessment (Supplementary Table 2).

#### Open-response feedback on lecture recordings

The final question on the survey was to ask the students to add any additional comments or feedback they had on the use of lecture recordings as a learning tool. In 2017, 9 of the 32 students completing the survey, and in 2018, 11 of the 41 students completing made additional comments/feedback. All (20) responses were positive, and the most common theme for accessing lecture recordings was ‘flexibility’ (e.g. when watched, ability to speed up or revisit) in 11 (55%) of comments. Examples included:

*Lecture recordings are awesome! When they don't work, I struggle.I like listening, and downloading and using VLC allows me flexibility of when l listen. I can speed the lectures up so they are quicker. And if I need to pause or go over anything I can.*

*I think they are great. I hate face-to-face lectures, as I cannot concentrate during them, especially when they are early in the morning. I like the flexibility of being able to watch the lectures at a time that is convenient for me, being able to speed them up to maximise efficiency, and being able to pause/rewind and write notes.*

*Lecture recording is the best tool for me and it is too convenient. Any time I can have access and listen, especially in the evening time. I listen to lecture recording mostly at night even if I attended. The Endocrinology unit is very big with too dense information. Its not easy to pick them up just by attending the lecture. Therefore, listening at relaxed time improve my understanding. I understand more better than attending. I personally can do go to work or drop my son to his school at 9 am not able to attend the lecture. When it uploaded, than its time for me to relax sitting somewhere even out of library with my own device and listen.*

*I use lecture recordings to be able to pause and rewatch difficult concepts and take my time on them. It also allows me to watch small 20m snippets on the bus or when walking.*

The second most common theme was ‘usefulness’ in 5 (25%) of comments. Examples include:

*I find the lecture recordings very useful, in particularly for those whose English is second language, so they have the opportunity to repeat the player if there is something unclear in the pronunciation of lecturer.*

*I find lecture recordings useful when I am unable to attend lectures. The lecture itself adds another layer on top of the notes and is useful for putting information learnt into context.*

*They are an important revision tool, which clarifies the content beyond the slides alone, and are especially useful when the lecturer chooses to add information not on the slides such as upcoming assessment updates, and practical information.*

Two students commented that they struggled when the recordings were not available/working, and another two students reported using them as a cramming/revision tool.

## Discussion

The main findings this study of lecture attendance and lecture recordings usage in two cohorts of third year undergraduate medical laboratory science students in a Diagnostic Endocrinology course were (i) lecture attendance by students was low, (ii) there was no association between lecture attendance and academic outcomes, (iii) there was no association between accessing lecture recordings and academic outcomes.

### Lecture attendance

Lecture attendance reported in previous studies of students in undergraduate biological sciences is generally higher than in this study, where the average percentage lecture attendance/student was 39% in 2017 and declined to 27% in 2018, despite the course being delivered similarly in both years. For the biological sciences courses where students had access to lecture recordings, higher attendance has been reported in most studies reporting on the association between lecture attendance and academic outcomes, despite the values having been calculated in a variety of ways. The values are 79% [10] or 91% [36] of students attending all lectures, 87% of students attending ≥75% of lectures [18], 90% of students attending ≥70% of lectures [12], 88% of students attending ≥56% of lectures [37], average attendance was 73% [15] or ranged from 20 to 90% in four courses [33] or from 58 to 95% in six courses [30]. The exceptions are a study reporting a similar attendance to the present study of 39% [38], and a recent study reporting that medical students only attended 24% of lectures in two courses [39].

One possibility for the lower attendance by students of medical laboratory science is that they attend less lectures than other biological science students, but this does not seem to be the case, as attendance in the biological sciences is also low for nursing [19] and biomedical science [40] students at QUT. Another possibility is that the declining lecture attendance relates to the present era (2017 onwards) and is supported by the 2018 findings from a USA medical college that only 24% of students attended lectures [41].

### Lecture attendance and academic outcomes

The present study shows that with the availability of lecture recordings, lecture attendance is not associated with better academic outcomes for students in a medical laboratory science course, and this is supported by some but not all previous studies. Thus, amongst students studying the biological sciences with access to lecture recordings, either no association between lecture attendance and academic outcomes ([18, 23, 36, 39] three of four courses [33]) or a positive association ([10, 12, 15, 18, 19, 37] one courses of four studied [33]) has been reported.

Bias is a possible reason for the discrepancy between studies of the relationship between lecture attendance and academic outcomes. For instance, in most of the studies of students studying the biological sciences, self-reported recall/surveys are the measure of attendance [10, 18, 23, 33, 37, 38] and, this may have subjected the results to both recall (not remembering correctly) or non-response bias. Thus, it is possible that students who attend lectures are more likely to participate and complete surveys than those that do not, the non-responders. There was no association between lecture attendance and academic outcomes in the present study in both the attendance sign-in component (90% participation rate) and survey (64% participation rate). Thus, it seems unlikely that the finding in this study of no association between lecture attendance and academic outcomes is biased by the manner the data was collected i.e. attendance register vs self-report.

The availability of lecture recordings may reduce the chance of there being a positive association between lecture attendance and academic outcomes for biological science students. In the absence of the reported availability of lecture recordings, a positive association was shown for many courses [3–9, 11, 13, 14, 16] but at least one course [22] has not shown an association. In the presence of lecture recordings, a positive association was also observed in several biological science courses ([12, 32, 34, 36, 37, 39] and for one in four courses studied by Davis et al. [33]) but not others ([23, 36], and three courses studied by Davis et al. [33]). A major limitation to this possibility, is that the comparison is between studies reporting and not reporting the availability of lecture recordings, which may not be equivalent to studies of courses that have or do not have lecture recordings available but have not reported on this.

Another possible reason for the discrepancy between this study showing no association and previous studies showing a positive relationship between lecture attendance and academic outcomes is that this study is considering much lower levels of lecture attendance to academic outcomes than most previous studies. Other studies with low levels of lecture attendance (39% [38], 24% [39]) have also shown no association between lecture attendance and academic outcomes.

Another possibility for the lack of association between lecture attendance and overall mark is that the overall mark is not solely dependent on knowledge of lecture content. Thus, only 40% of marks were allocated to the final examination, which was restricted to lecture content, and the other 60% of marks were related to ongoing assessment (laboratories/assignment). However, there was also no association between lecture attendance and the examination or ongoing assessment, which suggests that lecture attendance was not a determinant of either overall or examination mark.

### Rates of self-reported lecture recordings access

In this study, the number of students self-reporting accessing the lecture recordings most weeks or sometimes was high; 67%. This is in the range of self-reported lecture recording access by medical students; ~ 80% [30], ~ 50% [41].

### Lecture recordings and academic outcomes

Previous studies have variously reported no association, a positive association or a negative association between accessing lecture recordings and academic outcomes. The reasons for this variation in findings are unclear. The present study showed that self-reported accessing of lecture recordings was not associated with academic outcomes in a biochemistry course, and is supported by other studies showing no association in a BSc course [25], medical science courses [27, 28], pharmacy students [29], for seven of eight courses undertaken by medical students [30] and six of seven courses undertaken by dental students [23]. However, the present finding contrasts with a recent study showing a positive association between accessing lecture recordings and academic outcomes for nursing students undertaking a course in anatomy, physiology and microbiology [26]. This recent study differs from ours, in that the nursing students also had access to other online resources i.e. concept clips and an interactive anatomy atlas, which may have contributed to the positive association between the access to lecture recordings and academic outcomes [26].

The finding of no association between self-reported access to lecture recordings also contrasts with other studies of students showing a negative association for students studying the biological sciences (medical students [31], nursing students [32]). Other studies have showed that when students used lecture recordings in preference to attending pharmacology lectures, their academic outcomes were lower [10, 30]. It seems unlikely that accessing lecture recordings per se has a harmful effect on academic outcomes. The authors of these two studies have not given this, or any other, reason for the negative association. Others have suggested that the high use of lecture recordings represents ‘cramming’ by the weaker students, and this is responsible for the negative association [32]. This is supported by a previous study describing how the lower achieving students in a faculty of health were accessing lecture recording more often than high achievers [42]. However, in the present study, only 8% of the non-attending students reported ‘cramming’ as one of their reasons for accessing lecture recordings, and both students obtained higher than average overall marks.

### Student responses regarding lecture attendance

Many of the student responses to the survey indicate that they are using the course resources to their advantage, and as academics would anticipate. Thus, it was reassuring that most students chose to attend lectures because they ‘think they learn more by attending’ and to access lecture recordings to ‘reinforce and revise concepts on a regular basis’. The additional comments and feedback on lecture recordings also showed that the students had a good understanding of how to use the flexibility of these.

However, it was concerning that ≥50% of attending students did so because they were ‘concerned that recordings may not be complete or the technology for recording may fail’. As there were no issues with the actual lecture recordings during this study, these concerns may relate to previous experiences or experiences in other courses. To alleviate these student concerns, universities need to ensure that their lecture recording systems are always working, and that their lecturers are using these systems correctly.

At QUT, lectures are scheduled between 8 am and 10 pm. The lectures for this Diagnostic Endocrinology course were at 8 am, and this was unpopular with the students, and dominated the reasons for not attending lectures in the survey. A previous study has also reported that 8 am lectures are not popular with biochemistry students [14]. At QUT, there can be large gaps between lectures for individual cohorts of students, and this was indicated by students who did not attend lectures stating that it was ‘too far to travel for lectures’ and ‘having too few timetabled classes that day and didn’t want to come for just those’. This suggests that it may be possible to improve attendance rates at lectures by timetabling lectures later, and timetabling lectures and laboratories/tutorials for individual cohorts together.

Although a high percentage (≥ 95%) of students were successful in passing this Diagnostic Endocrinology course, their overall marks only averaged 69% ± 10 and were lower in examinations; 51% ± 19 (102). Thus, it is possible to speculate that many of the students would have performed better if they had attended more lectures or accessed more lecture recordings.

### Student responses regarding use of lecture slides (PowerPoints)

After finding out in 2017 that students passed the course in Diagnostic Endocrinology despite being poor attenders of lectures and accessing the lecture recordings sparingly, we considered whether other factors may have contributed to this success. We considered whether using lecture slides, independently of viewing lectures, may have contributed to the academic success of students, and therefore added an additional question to the survey in 2018 to address this issue. The answer to this question indicated that lecture slides were used extensively to study prior to assessment or examination, but there was no difference in academic outcomes for students who used lecture slides in this way or not.

### Strengths

One of the strengths of this study is the high participation rates for both the sign-in (90%) and survey (64%). Consent was sought separately for these components, as it was a concern that this may be lower for the survey than the sign-in, which was the case. However, the rate of response to the survey was still good. Another strength of the study was being able to show that the overall marks and lecture attendance of students was similar for participants in the sign-in and survey study.

### Limitations

There are several limitations to this study. Firstly, it is a relatively small study of two groups of students (medical laboratory science students) studying Diagnostic Endocrinology. Secondly, the introduction and discussion are limited to students studying biological and health sciences. Thus, the results are limited to biological science students and may differ from those for students studying other disciplines. Thirdly, the data on accessing lecture recordings by the students is self-reported from the survey. At the study site, quantitative data on accessing lecture recordings by students who directly access the recordings from Blackboard/ the learning management system is available, but not for those students who downloaded the lecture recordings prior to accessing them. As some students indicated that they downloaded lectures, it was decided not to use this data.

## Conclusions

Universities should endeavour to timetable lectures between 9 am and 4 pm to encourage students to attend and ensure that lecture recordings are consistently available. From this study, it is not possible to determine which aspects of the course are important for positive academic outcomes. Thus, it does not seem that either lecture attendance or accessing lecture recordings alone are major determinants of academic outcomes for most students. Rather it is possible, considering the data from the attendance register and survey, that a choice by the students of lecture attendance, lecture recordings and slides, used independently of lecture recordings, are used individually by students in achieving their academic success. This implies that academic staff should ensure that a mix of resources are made available to students.

## Supplementary information

**Additional file 1.** LECTURE ATTENDANCE and RECORDING SURVEY.

**Additional file 2.** Additional questions asked in 2018.

**Additional file 3: Supplementary Table 1.** Lecture attendance and academic outcomes for students in a medical laboratory science course from sign-in.

**Additional file 4: Supplementary Table 2.** Pearson’s correlation (r) of academic outcomes vs lecture attendance, lecture recording usage, lecture slide use and engagement from survey.

## Data Availability

The datasets used and/or analysed during the current study are available from the corresponding author on reasonable request.

## Abbreviations

QUT: Queensland University of Technology
IDs: student numbers
r values: Pearson’s correlation coefficients
SD: standard deviation of mean
