# Supplementary material for: No apparent association between lecture attendance or accessing lecture recordings and academic outcomes in a medical laboratory science course
Source: BMC Med Educ. 2020 Jun 30;20:207. doi: 10.1186/s12909-020-02066-9 (PMC7329538; doi:10.1186/s12909-020-02066-9)
Supplement: Supplementary file 1 — Additional file 1. LECTURE ATTENDANCE and RECORDING SURVEY. [file 12909_2020_2066_MOESM1_ESM.docx]

|  |
| --- |
|  |
|  |
| Supplementary online information  [LECTURE ATTENDANCE and RECORDING SURVEY](https://docs.google.com/forms/d/e/1FAIpQLSc7-jsaZJ_caPLDu7W448IcjYTrVCpPVWcExIrbsAeoGsu1xg/viewform?c=0&w=1&includes_info_params=true&usp=mail_form_link) |
|  |
| Lecture recording, as full audio and screen capture presentations, are a relatively recent introduction. Little is known about the factors determining whether students attend lectures, use lecture recordings, and their relationship. As part of our ongoing research we are investigating this, and you can help us by filling out this survey.  Participation in the survey implies consent for us to link this to lecture attendance records and grades (if previously agreed to). |
|  |
| 1. **Please enter your name**      1. **Please enter your student ID**      1. **Which of these units are you enrolled in for Semester 2 2017?**    LSB111 Understanding Disease Concepts   LSB425 Quality and Analysis in Clinical Pathology   LSB625 Clinical Endocrinology   LQB600 Physiological Basis of Pharmacology   1. **Do you identify as male or female?**    Male   Female   Prefer not to say   1. **Which age group were you when you started your present course?**    Under 21 years of age   21 years of age or older   1. **Is English your first language?**    Yes   No   1. **Are you an international student?**  - Yes - No  1. **Do you have a job?**    Yes   No   1. **If you have a job, how many hours per week do you work?**    10 hours or less   More than 10 but less than 20 hours   20 hours or more   1. **What is your study load?**    1-2 units   3 or more units   1. **Did you attend scheduled lectures?**    YES - most weeks. Go to Question 12   Sometimes - go to Question 12   NO - go to Question 16   1. **Why did you choose to attend lectures?**   Select all options that apply.   I think I learn more by attending   I think my results will be better if I attend   It allows for interaction with unit staff and/or students   To catch up with my friends   It is good to be seen to be attending   I am concerned that recordings may not be complete or the technology for recording may fail   I like to see the lecturer's gestures and expressions   Other:    1. **If you attended lectures did you also access the recorded lectures on Blackboard?**    Yes - most recordings. Go to Question 14   Sometimes. Go to Question 14   No - go Question 18   1. **Why did you access the recordings as well as attend the lectures?**   Select all options that apply.   Catch up on the lectures I missed   Reinforce and revise concepts on a regular basis   Revise lecture concepts for assessment purposes   Clarify difficult concepts   I find it hard to concentrate in the lecture theatre   Other:    1. **Did you find it useful to hear and see the lecture content again after attending scheduled lecture?**   Select the relevant option. 1 = not useful at all, 2 = quite useful e.g. for study and 3 = very useful for study and increasing understanding of concepts   \|  \| 1 \| 2 \| 3 \|  \| \| --- \| --- \| --- \| --- \| --- \| \|  \|  \|  \|  \|  \|   **16. If you did not attend lectures, why not?**  Select all options that apply.   Work commitments made it difficult to get to lectures   Other personal commitments made it difficult to get to lectures   I had too few timetabled classes that day and didn’t want to come in for just those   I don't like the lecture theatre environment   I don't consider the lecturer adds to the material given on the PowerPoints   I didn't like the lecture time - it was too early   I didn't like the lecture time - it was too late in the day   Too far to travel   Timetable clash   Placement commitments   When assessment tasks were due they took preference over lecture attendance  Other:   **17. If you did not attend lectures, did you listen to the recordings?**  Select all options that apply.   Yes, weekly - go to Question 18   Yes, every few weeks – go to Question 18   Yes when completing an assessment item - go to Question 18   Yes at the end of semester before the final theory exam - go to Question 18   No, – go to Question 19   1. **If you did not attend lectures, why did you access the recordings instead of attending lectures?**   Select all options that apply.   Work commitments made it difficult to get to lectures   Other personal commitments made it difficult to get to lectures   I prefer the flexibility of the online recordings   I had too few timetabled classes that day and didn’t want to come in for just those   I don’t like the lecture theatre environment   Prefer online learning environments   I don't consider the lecturer adds to the material given on the PowerPoints   I didn't like the lecture time - it was too early   I didn't like the lecture time - it was too late in the day   Too far to travel for lectures   I chose to rely on cramming the lecture material at the end of semester using the recordings   Timetable clash   Placement commitments   When assessment tasks were due they took preference over lecture attendance   1. **If you did not access the recordings or attend the lectures, which resources did you use for assessment purposes?**   Select all options that apply.   Teaching resources on Blackboard e.g. PowerPoint slides   Other students' notes   Reference books   Practical information and reports   Nothing – I winged the assessment  Other:    1. **In addition to lectures and practicals, how many hours did you spend on this unit in a week when not working on major assessment?**    No additional hours   Less than 2 hours   2 to 4 hours   More than 4 and less than 8   8 or more hours     1. **In addition to lectures and practicals, how many hours did you spend on this unit in a week when you were working on major assessment?**    No additional hours   Less than 2 hours   2 to 4 hours   More than 4 and less than 8 hours   8 or more hours   1. **Please include any additional comments or feedback you have on the use of lecture recordings as a learning tool.**    |
|  |
