# Supplementary material for: No apparent association between lecture attendance or accessing lecture recordings and academic outcomes in a medical laboratory science course
Source: BMC Med Educ. 2020 Jun 30;20:207. doi: 10.1186/s12909-020-02066-9 (PMC7329538; doi:10.1186/s12909-020-02066-9)
Supplement: Supplementary file 2 — Additional file 2. Additional questions asked in 2018. [file 12909_2020_2066_MOESM2_ESM.docx]

Supplementary online information 2

Additional questions asked in 2018

**14. Other than as part of lecture recordings, do you use hard copies of the provided Powerpoint slides or Powerpoint slides online?**

- Yes - Go to Question 15
- No – Go Question 16

**15. How do you use the PowerPoint slides?**

- To study prior to lectures
- To study during lectures
- To study instead of attending lectures
- To study instead of listening to lecture recordings
- To study prior to assessment or examinations
