# Supplementary material for: No apparent association between lecture attendance or accessing lecture recordings and academic outcomes in a medical laboratory science course
Source: BMC Med Educ. 2020 Jun 30;20:207. doi: 10.1186/s12909-020-02066-9 (PMC7329538; doi:10.1186/s12909-020-02066-9)
Supplement: Supplementary file 3 — Additional file 3: Supplementary Table 1. Lecture attendance and academic outcomes for students in a medical laboratory science course from sign-in. [file 12909_2020_2066_MOESM3_ESM.docx]

**Supplementary Table 1.** **Lecture attendance and academic outcomes for students in a medical laboratory science course from sign-in**

| **Attendance** | **33% cut-off** | | **50% cut-off** | | **70% cut-off** | | **None** | **At least 1** |  |
| --- | --- | --- | --- | --- | --- | --- | --- | --- | --- |
|  | **<** | **≥** | **<** | **≥** | **<** | **≥** |  |  | **All students** |
| **2017** |  |  |  |  |  |  |  |  |  |
| **Number of students** | 18 | 23 | 25 | 16 | 33 | 8 | 11 | 30 | 41 |
| **Overall mark** | 66 ± 12 | 71 ± 11 | 67 ± 12 | 73 ± 9 | 68 ± 11 | 76 ± 10 | 68 ± 14 | 69 ± 11 | 69 ± 11 |
| **Examination** | 50 ± 19 | 58 ± 18 | 49 ± 18 | 60 ± 18 | 51 ± 17 | 64 ± 21 | 46 ± 14 | 54 ± 16 | 54 ± 19 |
| **Ongoing assessment** | 78 ± 9 | 80 ± 7 | 81 ± 6 | 78 ± 9 | 78 ± 8 | 83 ± 6 | 79 ± 6 | 79 ± 8 | 79 ± 8 |
| **2018** |  |  |  |  |  |  |  |  |  |
| **Number of students** | 40 | 24 | 49 | 15 | 56 | 8 | 28 | 26 | 64 |
| **Overall mark** | 66 ± 11 | 68 ± 10 | 67 ± 5 | 65 ± 20 | 67 ± 11 | 65 ± 5 | 66 ± 10 | 67 ± 11 | 67 ± 10 |
| **Examination** | 42 ± 21 | 51 ± 19 | 46 ± 11 | 44 ± 22 | 46 ± 21 | 45 ± 13 | 43 ± 19 | 48 ± 21 | 46 ± 20 |
| **Ongoing assessment** | 81 ± 6 | 80 ± 5 | 81 ± 3 | 79 ± 10 | 81 ± 6 | 78 ± 3 | 82 ± 6 | 80 ± 6 | 81 ± 6 |

Each value is mean ± SD, all P values were > 0.05 by Student’s unpaired t-test
