# Supplementary material for: No apparent association between lecture attendance or accessing lecture recordings and academic outcomes in a medical laboratory science course
Source: BMC Med Educ. 2020 Jun 30;20:207. doi: 10.1186/s12909-020-02066-9 (PMC7329538; doi:10.1186/s12909-020-02066-9)
Supplement: Supplementary file 4 — Additional file 4: Supplementary Table 2. Pearson’s correlation (r) of academic outcomes vs lecture attendance, lecture recording usage, lecture slide use and engagement from survey. [file 12909_2020_2066_MOESM4_ESM.docx]

**Supplementary Table 2 Pearson’s correlation (r) of academic outcomes vs lecture attendance, lecture recording usage, lecture slide use and engagement from survey**

| **Academic outcome** | **Lecture attendance** | **Lecture recording usage** | **Lecture attendance and lecture recording usage** | **Lecture slide use** | **Engagement** |
| --- | --- | --- | --- | --- | --- |
| **2017** (n = 31) |  |  |  |  |  |
| Overall mark | 0.177 | 0.014 | 0.144 |  |  |
| Examination lecture content | 0.169 | 0.290 | 0.304 |  |  |
| Ongoing assessment | -0.015 | 0.302 | 0.170 |  |  |
| **2018** (n = 44) |  |  |  |  |  |
| Overall mark | -0.048 | -0.015 | -0.027 | -0.168 | -0.111 |
| Examination lecture content | -0.000 | 0.017 | 0.010 | -0.175 | -0.085 |
| Ongoing assessment | -0.204 | 0.072 | -0.110 | -0.119 | -0.152 |

Engagement is a combination of lecture attendance, lecture recording use and lecture slide use

All P values were > 0.05 by Student’s unpaired t-test

|  |
| --- |
|  |
|  |
|  |
